# Fostering behaviour change in farm veterinary practice: ‘champion’ goal-setting and implementation considerations for antimicrobial stewardship

**DOI:** 10.1093/jacamr/dlaf181

**Published:** 2025-10-24

**Authors:** A M Bard, G M Rees

**Affiliations:** Bristol Veterinary School, University of Bristol, Langford BS40 5DU, UK; Department of Life Sciences, School of Veterinary Science, Aberystwyth University, Aberystwyth SY23 3DA, UK

## Abstract

**Background and objectives:**

The use of antimicrobials in human and animal health care settings is considered a major driving force behind the emergence of antimicrobial resistance, encouraging a focus on evidence-based interventions aimed at promoting behaviours aligned with antimicrobial stewardship (AMS) ideals within both sectors. The Arwain complex intervention within Wales established peer networks of ‘Veterinary Prescribing Champions’ (VPCs) within and between Welsh veterinary practices to facilitate AMS training, support and activity. This study evaluates AMS goal setting and implementation within continuously engaged Arwain veterinary practices (*n* = 34) between 2020 and 2024.

**Methods:**

Descriptive quantitative analysis of Practice Action Plans (2020, 2024) and self-reported data on Action Plan implementation (2024), combined with qualitative analysis of participant interviews (2023) and participatory workshop feedback (2024) on challenges to implementing change, offer insight on the focus, achievements and implementation of AMS within the Arwain complex intervention.

**Results:**

VPCs focused on behaviour-led (practice team/farm client) and structural (farm/practice-focused) changes. All practices reported at least one change goal initiated, with the majority (73%) reporting at least one fully implemented change. AMS implementation challenges included practical and cultural considerations of veterinary practices, the complexities of delivering AMS within inter-professional teams, the situated complexity of AMS on farm and the geographic, economic, regulatory, epidemiological and attitudinal factors implicit in the practice ‘outer setting’.

**Conclusions:**

The Arwain complex intervention led to successful implementation of AMS changes across participating practices, with varying complexity, abstractness and completeness. Further research into the impact on antimicrobial use is needed to evaluate and inform future policy.

## Introduction

Antimicrobial resistance (AMR) is recognized as a cause of global One Health concern.^1^ The use of antimicrobials in human and animal health care settings are considered major driving forces behind the emergence of AMR,^2^ encouraging a focus across both professional landscapes on evidence-based interventions aimed at promoting antimicrobial stewardship (AMS) ideals i.e. the use and prescription of antimicrobials ‘in a way that ensures the availability of antimicrobials for individuals in the present day, as well as preserving antimicrobial effectiveness for current and future populations’.^3^

In the UK, veterinarians have a direct and crucial role in managing national livestock antimicrobial use (AMU)^4^ as the only legal prescriber of antimicrobials^5^ to farm animals. Veterinary prescribing is both a medical and economic practice,^6^ where professional jurisdiction over AMU is entwined with both an Aesculapian healing identity^7^ and the financial viability of practice operations (drug sales contribute significantly to veterinary practice revenues^6^). Veterinarians have significant autonomy in their antimicrobial prescribing^8^ and value this professional freedom,^9^ and whilst the UK operates under national AMR strategies, industry and associated stakeholders are largely responsible for defining and practically achieving strategy targets.^4^ The UK livestock industry has nevertheless made considerable progress in reducing, refining and replacing AMU over the past decade to have among the lowest antibiotic sales in Europe on a mg/kg basis,^10^ an outcome made more salient by the relative paucity of ‘top down’ regulatory interventions relative to other European nations.^4^

UK farm veterinarians however remain under increasing national pressure to achieve further progress in response to the threat of AMR.^11^ One evidence-based intervention approach to encouraging responsible AMU in the veterinary sector is via cultivating social support for veterinary clinicians, with Regan *et al*.^12^ highlighting the Arwain complex intervention within Wales^13^ as a successful example of this strategy. In this intervention, we established peer networks within and between Welsh veterinary practices to facilitate AMS training, support and activity and establish a community of practice—i.e. ‘a network made up of individuals and organizations that share an interest and practice, who come together to address a specific challenge, and further each other’s goals and objectives in a specific topic area’^14^—with this relational community engagement hypothesized to be a key ingredient of the intervention.^13^ Participants also adopted the role of veterinary prescribing ‘champions’ within their participating practices, a common implementation approach in healthcare interventions. A ‘champion’ assumes a position of leadership or advocacy for a specified intervention,^15^ with their presence or absence having the potential to mediate or moderate intervention outcomes.^16^ However, informed by Self-Determination Theory,^17^ the Arwain network ‘Veterinary Prescribing Champion’ (VPC) role was instead consciously designed to embody clinician autonomy: veterinary ‘champions’ acted as the *architects* of AMS change, rather than advocating for an externally imposed AMS intervention.^13^ Participatory interventions within health services suggest that where participants have autonomy over AMS activity this may be important for successful outcomes.^18,19^

Full details of the Arwain complex intervention design, recruitment, participant training and logic model can be found in Rees *et al*.^13^ Using both quantitative and qualitative data, this paper describes the AMS change planning, reported achievement and implementation experiences of participants to address three research questions; namely:

- Focus: when given autonomy over what and how AMS is implemented at a practice level, what change goals are chosen by participating veterinarians?
- Achievement: what level of success is witnessed over a three-year period, with regards to initiating and completing planned AMS change goals?
- Implementation experiences: what external challenges are reported by participants implementing change within the veterinary practice environment?

In doing so, this paper offers new insight on whether champion-led activity within communities of stewardship practice can lead to meaningful AMS behaviour change, and the complexities of the implementation setting that should be considered in AMS interventions of this kind.

## Materials and methods

### Ethics

This research was conducted in accordance with the Declaration of Helsinki and national and institutional standards. The Arwain Vet Cymru/Arwain DGC project was approved by the Ethics Committee of the University of Bristol Faculty of Health Sciences (ref 99522) and Aberystwyth University Ethics Board (ref 18404). Written informed consent was obtained for study involvement at the practice level and verbal consent at the participant level for contributions within the Arwain Network as the practice ‘VPC’.

### Study participants

**Figure 1.**
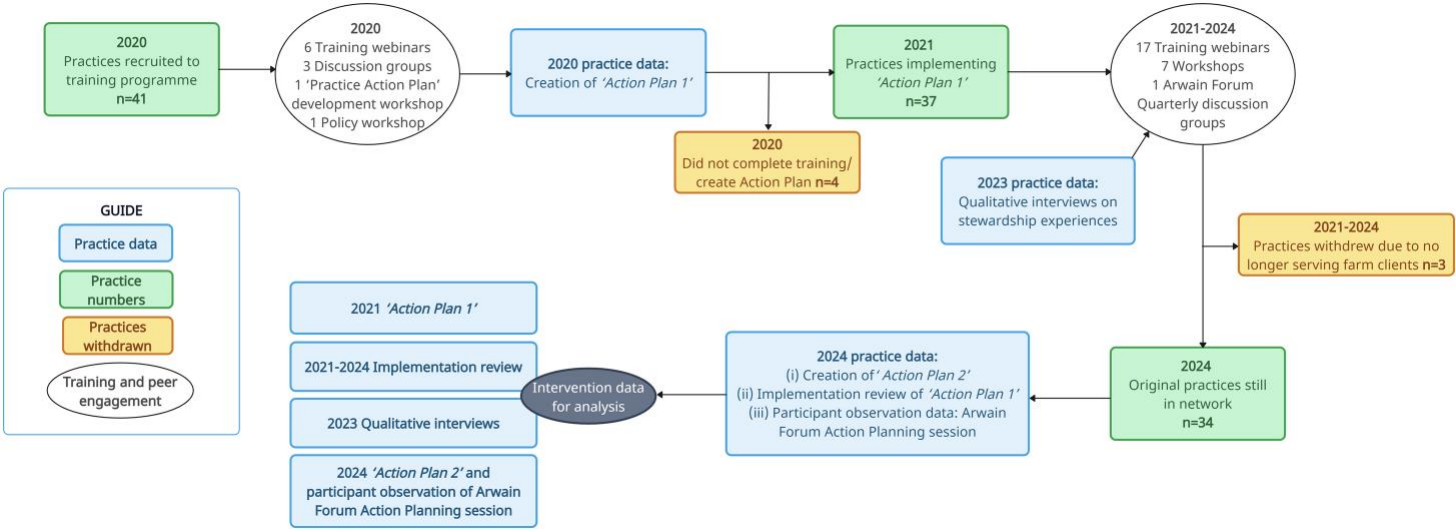
Practice recruitment, retention, data collection and AMS training/peer engagement activities within the Arwain Network.

A total of 41 veterinary practices across Wales were recruited to the Arwain Vet Cymru project from March 2020. Out of the 50 eligible Welsh practices involved in farm work at this time (defined as practices registered as delivering Government TB testing in cattle), nine did not take part in the programme (see Rees *et al*.^13^ for further information). Within these 41 recruited practices, each named a specific farm veterinarian who would represent the practice as their VPC. This individual was invited to attend a series of CPD webinars, discussion groups and workshops (Figure 1) to enhance their competence in AMS, build interpersonal relationships within the network and develop a bespoke AMS Action Plan for implementation within their practice. This complex intervention was theoretically grounded in Self-Determination Theory, with the intention of careful integration of participant motivational needs within these participatory and educational online activities.^13^

Following this period of skills training and relational development, practice veterinarians were required to submit a ‘Practice Action Plan’ indicating how they intended to reduce, rationalize and replace AMU to formalize their role as a ‘VPC’, representing their practice in the Arwain network and the Arwain network within their respective practices. Participants defined AMS change goals themselves, without influence or input by Arwain staff. Of the sample (*n* = 41), a total of 37 practices submitted an Action Plan. As of 2024 collection of intervention data (Figure 1), 34 of these practices were still active in the Arwain network (the remaining withdrew due to ineligibility: i.e. were no longer serving farm clients), which has continued as part of a wider, interdisciplinary project called Arwain DGC in Wales, United Kingdom and has since grown to *n* = 47 practices and *n* = 72 VPCs as of March 2025. Throughout the project, all VPCs were invited for optional check-ins and support with regards to their Action Plan delivery. This included more formal sessions—via Action Plan focused discussion group meetings (2021) and in-person peer-to-peer workshops on Action Planning and implementation (2022: external facilitator in both North/South Wales locations, 2024: AB and GR, Mid-Wales location)—in addition to more informal opportunities for implementation-focused discussion, regularly cultivated in wider AMS training and peer engagement activities (e.g. those focused on soft skills, species-or disease-specific ‘best practice’ AMU and the regulatory landscape of AMS: 2021–24).

### Data collection

#### 2020: Establishing AMS goals within the Arwain network

On joining the Arwain intervention in 2020—following a 3-h online Action Plan development workshop—VPCs were invited to submit ‘Practice Action Plan 1’ (Table 1,^20^ Figure 1) via email, indicating a practice commitment to implement specified change goals from January 2021 onwards. AMS Action Plans involved goal setting—an effective behaviour change technique^21^—via the common ‘SMART’ goal setting heuristic.^22^

**Table 1.** Written documentation collected within the Arwain complex intervention for practices continuously active in the network

Year	Documentation	Purpose	Questions asked of practice veterinary prescribing champion
2020	‘Practice Action Plan 1’	Establishes practice commitment to implement specific AMS change goals	Please briefly describe the change you plan to make in your practice: What elements of the RESET^a^ model does this use? Why did you choose to make these changes? What barriers might you need to overcome? (Specific) What specific actions will you take? (Measurable) How will you know if it works? (Achievable) How will you implement it? (Relevant) What difference will it make? (Time bound) When would you like to see improvement? (Assignable) Who needs to be involved to ensure success?
2024	‘Practice Action Plan 2’		
	Change goal ‘Implementation Review’	Ascertain level of implementation of specific AMS change goals within the practice	Categorize your AMS strategies by their level of implementation: *Total: this action has been implemented* *Patial: steps have been taken towards implementing this action* *None: no steps have been taken towards implementing this action*

^a^The RESET model, established by Lam *et al*.^20^ establishes important cues for changing human behaviour via rules and regulations, education, social pressure, economics and tools. This model was referenced in the Action Plan documentation following integration in participant AMS training experiences (i.e. discussion groups and webinar provision).

#### 2023: Exploring participant experiences within the Arwain network

VPCs were invited to take part in a qualitative interview on their participation in the Arwain network in 2023 (Figure 1) exploring their personal background, experience of the intervention, their AMS activities, perceptions of being a VPC and their desired support from the Arwain project [interview schedule: Supplementary Material 1 (available as Supplementary data at *JAC-AMR* Online)]. Given the professional demands of veterinary practice and availability for scheduling time for this activity, VPCs were given a choice in the interview length they could opt for (15, 30, 45 or 60 min), with the interviewer adopting variations of the interview schedule responsively to this specific scheduling request (time-related adjustments detailed in interview schedule: Supplementary Material 1). Interview data were transcribed *intelligent verbatim* by an external transcription organization.

#### 2024: Exploring participant experiences within the Arwain network and refocusing AMS goals

All VPCs were invited to an all-day in-person participatory meeting (May 2024, Llandrindod Wells, Wales: hereafter ‘Arwain Forum’: Figure 1) to connect with network peers and discuss their Practice Actions Plans, in addition to reviewing other core aspects of the Arwain complex intervention. Those attending the Arwain Forum took part in a 90-min ‘Action Plan: Review and Refocus’ session where participants were (i) invited to reflect on the implementation of their ‘Practice Action Plan 1’ change goals between 2020 and 2024, by individually reviewing and reporting their coded change goals as ‘full’, ‘partial’ or ‘no’ implementation’ (‘Implementation Review': Table 1); (ii) establish a renewed ‘Practice Action Plan 2’ for their AMS activity in the coming year (2024 AMS documentation: Table 1) and (iii) engage in facilitated small-group discussion and group feedback in plenary on ‘*what facilitates and hinders implementation of AMS change goals?’*. This session was facilitated by AB, with participant observation and note-taking by GR. *Network VPCs unable to attend the Arwain Forum in person were posted an ‘Action Plan Pack’, followed up with copies of the (i) and (ii) documentation by email, to allow them to complete the Action Planning task asynchronously*.

#### Demographic data

All VPCs were invited to complete a demographic survey hosted in Google Sheets between June and November 2024.

### Data analysis

#### Focus of AMS: practice action plans

*2020: ‘Practice Action Plan’ 1:* Practice Action Plan data were manually entered into Microsoft Excel, organized by practice. Data were reviewed and coded without *a priori* codes or categories established regarding AMS interventions; instead, AMS codes were created and added iteratively as practice data were reviewed, depending on the content and intention detailed within Action Planning documentation. This process resulted in a comprehensive list of codes reflecting VPC AMS intentions for their veterinary practice. Once complete, all codes were reviewed and organized into core AMS themes and subthemes to provide clarity on the focus and intention of the AMS change goals: hereafter this summary of codes is described as Code Book 2020. Descriptive data reflecting the number and types of AMS intentions detailed by VPCs were collated using Microsoft Excel.

*2024: ‘Practice Action Plan 2’:* Practice Action Plan data were manually entered into Microsoft Excel organized by practice, with data reviewed and coded with Code Book 2020 as a framework. Where novel AMS intentions were detailed by VPCs that were not yet captured within Code Book 2020, additional AMS codes were created and added iteratively as practice data were reviewed, resulting in an updated Code Book for 2024. Descriptive data reflecting the number and types of AMS initiatives were collated using Microsoft Excel.

#### Achievement of AMS: ‘practice action plan 1’ implementation review

Implementation data were imported into Microsoft Excel and organized by practice according to Code Book 2020, allowing descriptive data on implementation to be produced reflecting the number and type of AMS change goals implemented within the Arwain network between 2020 and 24.

#### AMS implementation experiences: interview and workshop data

Qualitative coding of interview transcripts (*n* = 33) and workshop participant observation data were carried out deductively by AB, adopting the Consolidated Framework for Implementation Research (CFIR).^23^ The CFIR is one of the most highly cited frameworks within implementation science research, enabling assessment of contextual factors that act as active and dynamic forces on implementation efforts in real-world settings via 48 constructs and 19 subconstructs across 5 domains (‘inner setting’, ‘outer setting’, ‘individuals’, ‘innovation’ and ‘implementation process’).^23^

First, all constructs included in Damschroder *et al.’s*^23^ CFIR documentation ‘*Updated CFIR Domains and Constructs: Short Definitions and Detailed Descriptions’* were reviewed by AB. Second, through consideration of the research question driving this qualitative analysis—i.e. what external challenges are reported by VPCs implementing change within the veterinary practice setting?—three core implementation domains were chosen as foci that could effectively characterize participant experiences; (i) ‘individuals’ (i.e. the individuals involved with implementing, delivering and/or receiving VPC-led AMS); (ii) ‘inner setting’ (i.e. the veterinary practice setting in which AMS is implemented) and (iii) the ‘outer setting’ (the external contextual and environmental factors influencing AMS implementation). This considered and specific focus is supported by CFIR authors, who emphasize ‘*it is often not feasible to assess every construct in the framework; nor will every construct apply within every project’.*^23^ Third, the study CFIR analysis codebook was constructed by defining each CFIR domain with relevance to the Arwain intervention and replacing broad CFIR construct language with intervention-specific descriptors. Finally, a CFIR informed framework matrix for qualitative data analysis was established within Microsoft Excel, enabling deductive coding of interview transcripts and workshop participant observation notes according to ‘individuals’, ‘inner setting’ and ‘outer setting’ domain constructs; challenges associated with ‘individuals’ were summarized by ‘role’ subdomain.

## Results

### Participant data

Of the 34 practices still active in the Arwain Network in 2024, 33 returned a Practice Action Plan in 2024 (participant/practice demographics: Table 2). Of this 2024 Action Plan sample (*n* = 33), 30 also returned the AMS change goal Implementation Review. As such, data on diversity of changes represents a sample of *n* = 33, whilst achievement of AMS changes represents a sample of *n* = 30. Achievement data were collected either at the Arwain Forum 2024 (*n* = 21 practices) or received via email/post 2024 (*n* = 12 practices).

**Table 2.** Demographic information for Arwain practices and VPCs who responded to the intervention demographic survey

Veterinary practices^a^		
Characteristic	Number of practices	Percent of practices^b^
**Type**		
*Independent/locally owned*	*n* = 27	87%
*Corporate*	*n* = 4	13%
**Branches**		
*0*	*n* = 7	23%
*1–3*	*n* = 19	61%
*4+*	*n* = 5	16%
**Client base**		
*Mixed*	*n* = 17	55%
*Farm only*	*n* = 13	42%
*Farm and Equine*	*n* = 1	3%
**Number of vets full time**		
*0–5*	*n* = 13	42%
*6–10*	*n* = 8	26%
*11–15*	*n* = 8	26%
*>15*	*n* = 2	6%
**Number of vets part time^c^**		
*0–5*	*n* = 22	73%
*6–10*	*n* = 8	27%
*11–15*	*n* = 0	0%
*>15*	*n* = 0	0%
**Number of front of house staff:**		
*0–5*	*n* = 12	39%
*6–10*	*n* = 14	45%
*11–15*	*n* = 1	3%
*>15*	*n* = 4	13%
**Cattle herds served by practice^c^**		
*<100*	*n* = 9	30%
*101–200*	*n* = 7	23%
*201–300*	*n* = 7	23%
*301–400*	*n*-0	0%
*>401*	*n* = 7	23%

*VPCs* ^d^		
Characteristic	Number	Percent^b^
**Gender**		
Female	*n* = 17	55%
Male	*n* = 14	45%
**Position in practice**		
Business partner/director	*n* = 18	58%
Clinical director	*n* = 2	6%
Salaried assistant/associate	*n* = 12	39%
**Veterinary work**		
Farm vet	*n* = 12	39%
Cattle vet	*n* = 7	23%
Mixed practice vet	*n* = 12	39%

^a^Practice information was received from *n* = 31 of *n* = 33 Arwain practices.

^b^Rounded to nearest whole number.

^c^No characteristic information was received from *n* = 1 practice.

^d^VPC information was received for *n* = 31 of *n* = 36 Arwain practice veterinarians: during this intervention, *n* = 15 practices enrolled >1 ‘VPC’, *n* = 3 Practice Action Plans represent the input of two different veterinarians in 2020 and 2024.

Qualitative interview data were collected in March and April 2023, with *n* = 29 of the continuously engaged practices taking part (represented by *n* = 31 participants). Average interview length was 29.6 min (range 9.3–66.2 min). Qualitative participant observation data of the Arwain Forum 2024 represents the input of 29 Arwain practices and 31 participants.

### Focus of change goals

#### Characterization of AMS change goals

When given autonomy over how AMS is designed and implemented at a practice level, change goals (*n* = 156) chosen by VPCs for their practices could be represented under four themes (Table 3): those focused on Farm Client Behaviour (*n* = 23 codes), Farm Oversight (*n* = 15 codes), Practice Team Behaviour (*n* = 26 codes) and Practice Oversight (*n* = 8 codes). Each code was allocated once within each Action Plan i.e. there was not repetition of code allocation within plans. VPC’s Action Plans represented between 1 and 12 distinct AMS codes in 2020 (Mean 4.7, Median 4) and between 1 and 8 distinct AMS codes in 2024 (Mean 2.9, Median 3). A total of 56 distinct change goal codes were allocated in 2020, compared to a total of 41 in 2024. VPCs defined change goals at varying levels of abstractness within Practice Action Plans, with change goals reflecting activities from the concrete (‘changing the dispensary to red/amber/green’) to the conceptual (‘be more proactive with preventative disease strategies’). Examples of coded strategies are provided in Supplementary Material 2.

**Table 3.** Themes, categories and codes representing Practice Action Plan change goal foci in 2020 and 2024

				Change goal count	
Theme	Category	Category description	Code	2020	2024
**FARM CLIENT BEHAVIOUR** Strategies aiming to influence client AMU on farm, whether by shaping and scaffolding (i) client autonomous behaviour on farm in the absence of the vet or (ii) decision making in partnership with the vet when both are on farm	Education and training	Educational activities carried out directly with the client(s) with the aim of sharing/exchanging information and upskilling	Training/education: delivery context not specified	3	4
			Training/education: within HHP	5	2
			Training/education: within individual consultations (e.g. during TB tests, lambing)	3	1
			Training/education: standalone event (e.g. workshop)	7	9
			Explore client or vet interest in provision of AMU discussion/farm client meeting groups	0	1
	Access to information	Increasing the availability and visibility of information freely available to clients relating to AMR/AMU	Disseminating information via social media	3	0
			Disseminating information via agricultural shows	0	1
			Disseminating information via practice noticeboard display/posters	1	0
			Disseminating information via newsletter/leaflet	7	7
			Disseminating information via text messages	1	0
			Disseminating information via marketing infographics	1	0
	Refinement of AMU	In partnership with the vet, adjusting client access to and use of AMs to encourage more responsible use	Increase diagnostic use	2	1
			Reduce/stop prophylaxis	4	0
			Reduce injectable antimicrobials	1	0
	Alternatives to AMU	In partnership with the vet, encouraging behaviours in place of AMU when appropriate	Use mineral spray before antibiotics	0	1
			Increase use of NSAIDs	2	0
	Prevention	Working with the client to reduce their need for AMs on farm	Improve on-farm hygiene	1	0
			Encourage a more proactive/preventative approach on farm	6	4
			Increase farm client use of vaccinations	5	1
	Guidance/instruction	Scaffolding client behaviour to encourage responsible AMU	Improve antibiotic course compliance	1	0
			Distributing clinical guidelines/Arwain best practice guidelines	0	3
			Clients to have protocols for common conditions	1	0
			Encourage clients to improve their record keeping	1	0
**OVERSIGHT OF FARM AMU** Pursuing an effective working knowledge of farm management and/or AMU and using this knowledge/data in veterinary advice, communication and decision making	Monitoring	Gathering detailed data on how and whether AMs and AMU guidance are being used on farms	Monitor farm AMU (e.g. through practice management system, FAWL)	6	2
			Follow up on the implementation of vet recommendations within FAWL	0	1
			Ensure HHP protocols are in place and in use	0	1
			Gather mg/kg data on farms	1	0
	Auditing	Structured review of AMU or storage	Check/audit medicine cabinets on farm	5	0
			Improve AMU auditing	1	0
	Collating data	Bringing together veterinary data to inform, structure or influence discussions with the client regarding AMU	Collate data prior to HHP to support discussion	0	1
			Carry out prescriptions review within HHP (can include review of off label use)	1	1
			Collate farm AMU as a graphical educational tool for farmers	1	0
			Use costings and KPIs to motivate preventative client approach	1	0
			Medicine budgeting	4	2
			Benchmarking farmer AMU	5	2
	Targeting specific users	Using veterinary knowledge and data to identify specific clients requiring further attention	Target poor performing farms (identify through comparing health KPIs)	1	0
			Target high users on farms (e.g. interventions, education, new monitoring)	4	4
			Target non- farm assured clients (e.g. to quantify AMU)	1	1
**PRACTICE TEAM BEHAVIOUR** Strategies aiming to shape and scaffold practice team advice giving, prescription and dispensing (specific to their role)	Training	Educational activities carried out within the practice team with the aim of sharing/exchanging information and upskilling	Training/education: veterinary support staff (e.g. dispensing to farm clients)	7	4
			Training/education: specifically younger and less experienced vets	1	1
			Training/education: vet team	6	4
			Individual or wider team member becoming a VPC within Arwain DGC/engaging with Arwain DGC training	0	2
			More meetings/CPD within the practice (whole team or target participants unspecified)	1	1
	Guidance/instruction	Scaffolding practice team behaviour to encourage responsible AMU	Prescribing protocols: develop closer dialogue/emphasize importance	1	0
			Ensure vet familiarity with contract and assurance requirements	0	1
			Ensure practice displays prescribing guidance (e.g. EMA categorization)	0	1
			Ensure implementation of prescribing guidance/SOPs	0	1
			Developing or consolidating prescribing or dispensing guidelines/SOPs for best practice	10	2
			Create a prescription book for Large Animal medicines	1	0
			Tighten up veterinary cascade	1	0
	Promoting united voice	Bringing the practice team together to foster collaboration and shared practice on AMU behaviours	Developing a united voice on prescribing practices though one-on-one/team discussions and meetings	8	9
	Prescribing/dispensing	Shaping how and whether AMs are provided to clients by the practice upon request/prescription	Rearrange the pharmacy: no organization system specified/not traffic light system	3	0
			Implement traffic light system in dispensary	3	1
			New front desk rules with regards to dispensing of medicines	6	3
			Review dispensing procedures (as per VMD standards)	0	1
	Refinement of AMU	Rules aiming to adjust practice team access to and use of AMs to encourage more responsible use	A prescribing visit requires AM review (not just on assured farms)	1	0
			Large AB volumes or multiple doses of long-acting macrolides are now not permitted without work to investigate/improve management	1	0
			Access to CIAs limited (e.g. rules constraining use such as ‘always amoxicillin/oxytet before macrolides/cephalosporins’, requirements for diagnostic testing first)	1	1
			Halt access to antibiotic drench (lambing)	1	0
			Removal of CIAs from practice	1	0
			Clients have approved AM lists/thresholds—so larger quantities or unlisted AMs require approval/clients have an approved specific usage over set time period	1	4
			Use of licensed drugs only	1	0
			AMs only prescribed after confirmed diagnosis	1	0
**OVERSIGHT OF PRACTICE AMU** Pursuing an effective working knowledge of practice and vet team AMU and using this knowledge/data in advice, communication and decision making	Monitoring	Gathering detailed data on how and whether AMs are being used within the practice	Use or invest in new software for AMU data analysis (e.g. VetIMPRESS/RoboVet)	4	0
			AMU calculation: create supporting documents for vets to fill in to support FAWL/HHP review	0	1
			Establish more complex PMS farm records to link to AMU (e.g. virtual medicines cabinet, detail on dispensing by vet, rationales for prescribing/dispensing)	5	5
	Auditing	Structured review of AMU/dispensing/prescription	Check and follow up on antibiotic sales/prescription activities	1	0
			Assign lead vets to farms for accountability	3	1
	Collating data	Bringing together veterinary data to inform, structure or influence discussions with practice team regarding AMU	Improve vet awareness of practice AMU	1	0
			Collate AM sales data	0	3
			Collate vaccine sales and uptake data	0	1

AM, antimicrobial; AMU, antimicrobial use; CIA, critically important antimicrobial; CPD, continuing professional development; EMA, European Medicines Agency; FAWL, Farm Assured Welsh Livestock assessment; HHP, herd health planning; NSAIDs, non-steroidal anti-inflammatory drugs; SOP, standard operating procedure; TB, Tuberculosis; VMD, Veterinary Medicines Directorate; VPC, Veterinary Prescribing Champion.

#### Comparing 2020 and 2024 AMS change goal foci

**Figure 2.**
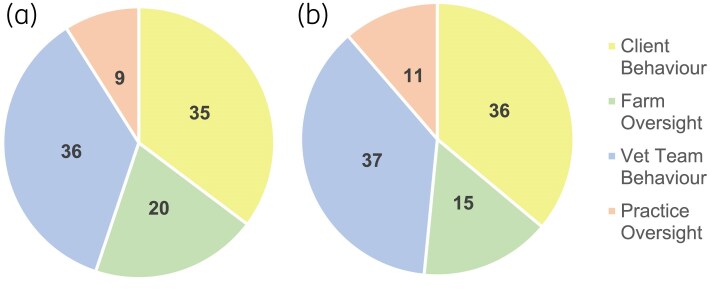
Practice Action Plan AMS change goals represented by theme in 2020 (*n* = 156: (a)) and 2024 (*n* = 97: (b)).

**Figure 3.**
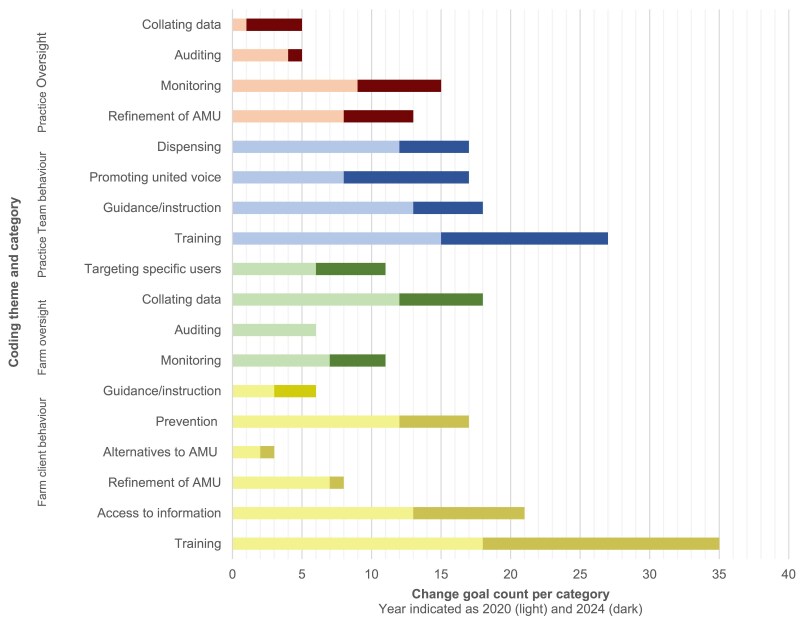
Count of Practice Action Plan AMS change goals established in 2020 (*n* = 156) and 2024 (*n* = 97).

Whilst the absolute number of AMS goals shifted from 2020 (*n* = 156) to 2024 (*n* = 97), change goal code attribution between the four themes remained comparable (Figure 2). Across all themes, AMS goals targeting education of clients and education of the practice team were the two most frequently attributed codes within 2020 and 2024 Action Plans. The popularity of change goal categories within overall themes was also comparable between 2020 and 2024 (Figure 3): further detailed information on change goal category rankings within themes is available in Supplementary Material 3.

### Implementation of AMS change goals

**Figure 4.**
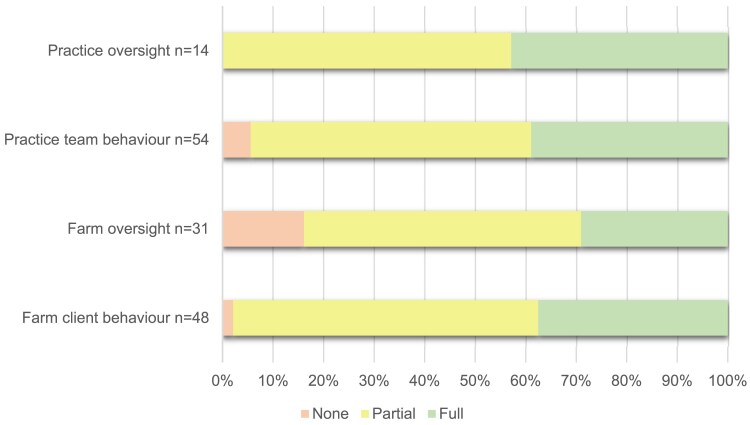
Reported implementation of all Practice Action Plan AMS change goals (*n* = 147).

**Figure 5.**
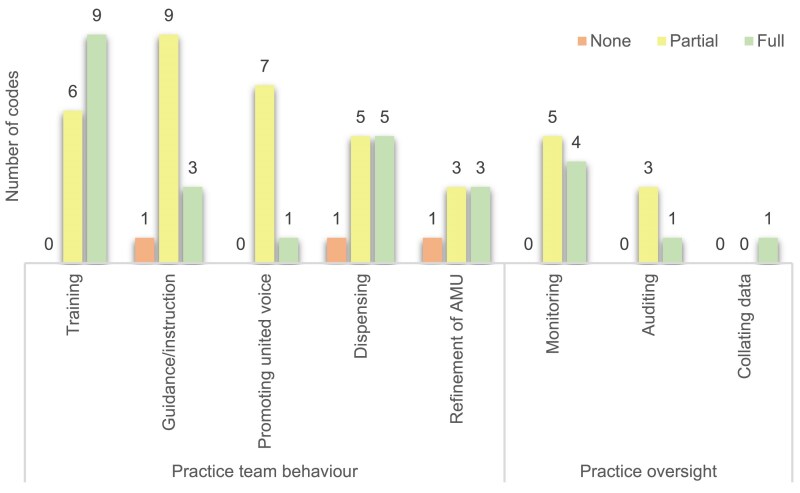
Reported implementation of practice focused Action Plan Goals (*n* = 68).

**Figure 6.**
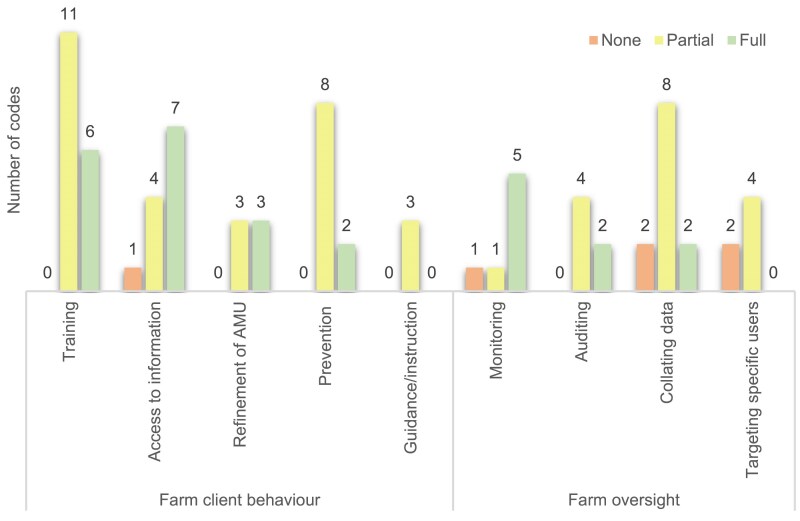
Reported implementation of farm focused Action Plan Goals (*n* = 79).

Between 2020 and 2024, 37% of planned changes (54/147) were implemented fully, 57% of planned changes were implemented partially (84/147) and 6% were not implemented (9/147). All practices had at least one change goal initiated (i.e. either ‘partially’ or ‘fully’ implemented: range 1–12), swith 73% of practices having at least one fully implemented change goal (range ‘fully’ implemented change goals: 1–6). When split by theme (Figure 4), change goal implementation ranged from 29% to 43% fully implemented, 55%–60% partially implemented and 0%–16% not implemented. Practice Oversight had all change goals reported as partially or fully implemented within practices. Farm Oversight had the highest percentage of change goals not implemented (16%). All change goals focused on training and education were either partially or fully implemented for both farm clients and veterinary practice teams (Figures 5 and 6).

### Challenges to implementing champion-led AMS

With regards to interacting with other individuals who may be involved in implementing, delivering and/or receiving champion-led AMS, challenges included prioritization and approval of AMS by high level leaders in the practice (e.g. directors), the variable knowledge, motivation, skills, responsibilities and experiences of others embroiled in practice AMS delivery (i.e. other farm veterinarians and front of house team members), in addition to navigating the complex situated considerations of AMS activity ‘recipients’; farm clients interacting with the practice for animal health service provision (Table 4).

**Table 4.** Implementation challenges reported by participants relating to individuals involved in implementing, delivering and/or receiving champion-led AMS, represented via the ‘roles’ subdomain of the Consolidated Framework for Implementation Research^23^

Construct	Challenges described
High level leaders in practice e.g. practice directors	Prioritization: if it's not on the priority list for those with influence in practice policy and action, it may be difficult to make changes Approval: waiting for ‘higher up’ approval can delay AMS action
Deliverers: practice veterinary team	Communication: deficits create difficulties in advising on change e.g. assuming farmer priorities and knowledge, not ‘pitching’ advice at the right level, appearing too directive/judgemental, engaging in agonistic interactions Individual variation: differing approaches due to age, experience, role (mixed/farm) or ‘personal nature’ can conflict with AMS Self-reflection: not all vets critically reflect on the AMR problem, which reduces motivation to change Ambivalence: there are considerable tensions in treating versus not treating (e.g. balancing animal health and farm economics, quick fix versus long term actions) Information deficits: vets must operate without perfect knowledge of client reality on farm, depending on client interactions Autonomy: It’s difficult to police vet behaviour—vets are ‘out there’ independently prescribing
Delivers: Front of House	Communication: changing dispensing practice can create difficult conversations with farm clients with established expectations Practice responsibilities: harder to prioritize CPD within practice to extend expertise around day-to-day work Information deficits: Front of House must operate without perfect knowledge of client reality on farm, depending on client interactions
Recipients: farm clients	Practical (finance, time): some practical challenges may not be possible to overcome to implement AMS change, or balancing future over current benefits challenging to envisage Change process: major change is often a slow process on farms Individual variation: differing approaches due to age, experience, ‘personal nature’ can conflict with AMS change Ambivalence: there are considerable tensions in treating versus not treating (e.g. balancing animal health and farm economics) Lack of awareness/knowledge: prescribing and dispensing rules, problem of AMR, AM types and uses (due to branding changes, or species dependent frequency of AMU) Established behaviours, expectations, norms: animal management processes, veterinary interactions and AMU access Farming industry/setting: difficult and stressful setting at times, which can build resistance to more external demands on activities Bureaucracy: AMS can feel like ‘box ticking’ if not carefully presented Complexity: managing farm health challenges can be complex Rapid pace of AMS change: large shifts in advice in just one decade Farm structure: as farms get bigger/more ‘professionalized’, they have more staff members and complex management to influence

AM, antimicrobial; AMR, antimicrobial resistance; AMS, antimicrobial stewardship; AMU, antimicrobial use; CPD, continuing professional development.

Within the ‘inner setting’ of the veterinary practice, VPCs implementing AMS had to navigate the challenges of conflicts with work infrastructure (staffing, season, practice activity), information technology infrastructure (the practice management system), conflicting elements of practice prescribing culture, minimal practice-wide connection opportunities, difficult communication within large practice teams, pressures to maintain the prescribing status quo (economic, professional responsibilities), difficulties in prioritization of AMS within practice ‘missions’ (i.e. their commitments, purpose, goals), limited funding support and variable access to AMS knowledge and information within the practice team (Table 5).

**Table 5.** ‘Inner setting’ implementation challenges experienced by participants, represented via constructs of the Consolidated Framework for Implementation Research^23^

CFIR construct	Challenges described
Work infrastructure	Staffing challenges to deliver AMS (rotas/being short staffed/large practice demands) Being responsive to emergencies/call outs/farm client needs Commitment to/demands of multiple projects Seasonal pressures of farm vet work
Information technology infrastructure	Practice management systems may not support accurate/nuanced analysis and reporting of farm AMU
Prescribing culture	Historical/established prescribing and dispensing practices e.g. Reliance of specific drugs in specific species Established client-‘front of house’ dispensing norms/practices Established veterinary prescribing norms/practices
Practice-team communication	Big practice teams make communication more difficult When teams get bigger the messaging gets ‘watered down’
Relational connections	It is a challenge to get entire practice teams in a room at the same time to discuss and agree change/develop team cohesion in AMS
Tension for change	*Economic* Status quo protects existing business derived from AMU sales Status quo ‘keeps clients happy’ and allied with the veterinary business through meeting their service expectations AMS aligned with expanding preventative role, vets report challenges charging appropriately for this type of advisory work *Professional responsibility* Fixing the ‘problem’ (e.g. via management and prevention) harder for vets than prescribing a ‘quick fix’ Potential consequences of not treating (e.g. animal welfare challenges, animal death, financial impact on farmer)
Mission alignment	AMS is not necessarily the top priority in veterinary practice
Funding	There is not additional funding available for many AMS change goals and farmers do not wish to increase their financial outlays
Access to knowledge and information	No mandatory CPD for ‘front of house’ team No mandatory CPD for veterinarians in responsible AMU practices

AMU, antimicrobial use; AMS, antimicrobial stewardship; CPD, continuing professional development.

In the outer practice setting, challenges to AMS included poor communication and lacking shared values with neighbouring vet practices, a regulatory landscape that may fail to enforce or undermine efforts towards responsible AMU in Wales, lack of accurate farm AMU metrics to facilitate AMS discussions with farmers, the lack of economic incentives to reduce AMU in the livestock setting, and the COVID-19 pandemic altering social interactions both within the veterinary practice team and with farming clientele for extended periods (Table 6). A novel construct was added to CFIR analysis to capture narratives on the practice geography, with regards to likelihood of local competition, the opportunities afforded by local farming systems, and potential for Arwain in-person engagement.

**Table 6.** ‘Outer setting’ implementation challenges experienced by participants, represented via constructs of the Consolidated Framework for Implementation Research^23^ in addition to the novel ‘practice geography’^a^ subdomain

Construct	Challenges
Practice geography^a^	*Competition*: Location in Wales felt to determine local competition environment and whether clients can source AMs elsewhere *Competition*: Not all vets/practices prescribing to farmers in Wales based here geographically, undercutting ‘united front’ of Arwain (e.g. border practices, visiting consultants) *Farming systems:* differing geographies support differing species, creating varying farm visit structures and AMU proclivities *Arwain:* national intervention necessitates extended journey times for in-person activity
Partnerships and connections	Lack of communication between veterinary practices with regards to clients and their prescribing relationships hinders transparency of farm AMU Neighbouring practices may not be in alignment with your AMS principles and may undermine practice AMS messaging
Policies and laws	Aspects of profession-level regulation felt to undermine AMS activities, messaging and Arwain aspirations; clients can legally source AMs from multiple practices and AMU data does not always represent this: *Subjectivity of ‘under care’ principles within RCVS guidance* *Lack of defined VCPR within RCVS guidance* *Lack of effective policing or penalties on illegal AMU activity* *No mandatory recording of AMU in a national database (e.g. linking holdings to veterinary practice AMU)* All Wales Arwain approach purely voluntary, ‘*no teeth’* to AMS efforts and activities HHP: obligations limited to those in farm assurance, which limits opportunities for AMS
Performance management *e.g. metrics, benchmarking*	Lack of accurate information on farm AMU to facilitate AMS discussion and engagement with farmers/foster AMU transparency, through: *Inaccurate medicine records on farm* *Imperfect farm assurance AMU recording systems* *No central, mandatory AMU database to encourage recording* *National use data (UK VARSS data reporting) too high level for utility* *No global AB identification system*
Local attitudes	AMS and AMR public facing communication may be comparably less prominent in farming/veterinary care than human health, meaning less potential for impact on local values and beliefs
Local conditions: economic	No economic incentives to reduce AMU (e.g. taxation)
Critical incidents	COVID-19 pandemic: limited or fundamentally altered social interactions within the veterinary practice team and with farming clientele for extended periods, impacting AMS activities (e.g. provision of in-person training, farm visit schedules and processes, appetite and energy for additional workload)

^a^AB, antibiotic; AMR, antimicrobial resistance; AMS, antimicrobial stewardship; AMU, antimicrobial use; HHP, herd health planning; RCVS, Royal College of Veterinary Surgeons; VARSS, Veterinary Antimicrobial Resistance and Sales Surveillance; VCPR, veterinarian-client-patient relationship.

## Discussion

This intervention adopted a ‘champion-led’ approach to facilitating AMS change within farm veterinary practice, with participants autonomously defining and enacting AMS change rather than implementing externally formulated AMS initiatives. Participants chose a variety of change goals oriented to both the veterinary practice team and their farm clientele, focused on behaviour-led (practice team or farm client) and structural (farm- or vet practice-focused) change. Coded intervention foci were diverse (Table 3) and included coverage of four of the seven core behaviour change intervention foci suggested by Regan *et al*.^12^ for promoting responsible farm AMU, via on-farm tools and prompts (e.g. ‘guidance/instruction’ category), social support for farmers (e.g. standalone farmer workshops), AMU monitoring (e.g. ‘collating data’ category) and social support for veterinarians (e.g. ‘promoting a united voice’ within the practice team). As such, the Arwain intervention appears to have successfully acted as a catalyst for evidence-based AMS activities within Welsh veterinary practice geographies.

The majority (73%) of practices reported at least one fully implemented change goal and a third of all planned change goals were reported as complete. Intended changes were successfully initiated (‘partial’ or ‘full’ implementation) for 94% of goals and all practices reported at least one change goal initiated. A high level of change initiation is pertinent to consider, given that whilst these change goals all contained specific information about end-states—i.e. the reference point towards which the AMS behaviour was directed—they were variable in their abstractness both within and between Practice Action Plans from the relatively concrete (e.g. achieving ‘*no critically important antibiotics on the shelf’*) to relatively more conceptual (e.g. achieving ‘*enhanced dialogue between farm teams on protocols’*).^24^ When considering change goals that are more abstract in nature, the ‘*less clear it will be as to what outcomes are acceptable as instances of goal attainment’*^25^ meaning reporting ‘full’ completion becomes a conceptually high bar. Indeed, the similar allocation of change goals within categories and themes between 2020 and 2024 could be reflective of many VPCs making only moderate adjustments when renewing their Action Plans, as a consequence of this difficulty in determining full goal attainment for the more conceptually defined AMS initiated.

**Figure 7.**
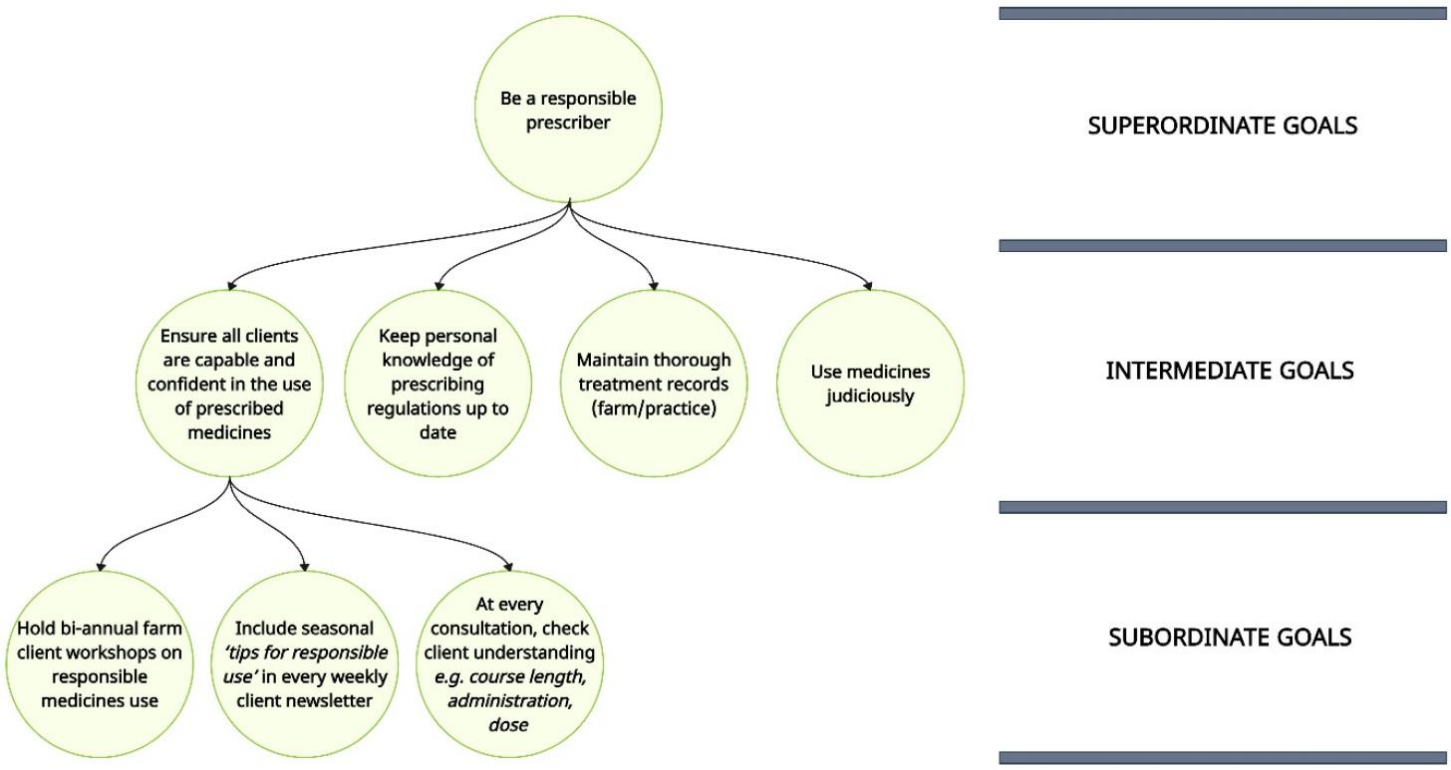
Schematic representation of potential veterinarian goals at three levels of abstraction (adapted from Hochli, Brügger and Messner^26^).

Theoretical and empirical work on goal setting suggests that establishing *interrelated* goals of varying abstractness when pursuing change can however support successful motivation and performance.^26^ Goal abstraction can be considered as a hierarchy,^27^ from subordinate (concrete: what to do, how to do it), to intermediate (general: the course of action) and superordinate (idealized principle: what is important) (e.g. Figure 7). The articulation and subsequent pursuit of interrelated goals that span this hierarchy is argued to increase long-term effort and motivation, inhibit competing goals, stimulate behavioural consistency and strengthen resilience.^26^ Future AMS interventions with autonomous champions could therefore benefit from a focus on strengthening the change goal articulation process as a behaviour change technique. By facilitating champions to consciously design AMS change goals that reflect these theoretical and empirical goal-setting principles, it could in turn foster greater participant motivation and performance, whilst supporting clarity in implementation reflection. Whilst the SMART heuristic was adopted within Arwain following the extensive representation of this framework in leading organizations and guidance nationally (e.g. NHS,^28^ Civil Service^29^), this framework is based on problematic scientific foundations; it did not originate from scientific theory or empirical evidence on goal-setting, suggesting more ‘*sophisticated, defensible and evidence-based’* goal-setting practices are indeed likely to enhance intervention success.^30^

Analysis of qualitative data highlighted a number of challenges to implementing AMS. Within the veterinary practice ‘inner setting’ these challenges reflected veterinary literature, where change is represented as engendering complex interactions between the inextricably linked factors of equipment and technology, working processes, organization structures and people.^31^ Participant narratives included practical considerations—such as work infrastructure (staffing, seasonality) or practice management IT systems—contrasted against the established ethos and culture of the veterinary practice. Conflicting ideals between ‘prescribing culture’ and AMS are recognized in human health literature, where physicians’ shared values may foster acceptance of antibiotic overuse, create avoidance of criticizing colleague’s antibiotic prescribing and establish competence as achieving clinical cure rather than minimizing adverse AMU impacts.^32^ Within the veterinary ‘inner setting’, the characterization of prescribing as both a medical and economic practice^6^ was reflected. VPCs narratives highlighted business needs (e.g. desires not to lose clients, to protect AMU sales, to charge appropriately for veterinary time) alongside professional responsibilities to ‘fix’ disease challenges and avoid negative consequences. In turn, these needs and responsibilities may support a practice prescribing culture in conflict with AMS ideals.

With regards to the individuals involved in AMS delivery, participant challenges included engagement with other veterinarians in the team, individuals in positions of authority/decision making in the practice and staff considered ‘front of house’; i.e. involved in the routine ordering, stock taking and dispensing of antimicrobials, who act as gatekeepers of antimicrobials and play a pivotal role in the flow of antimicrobials onto farms.^33^ In this way, numerous practice stakeholders were seen as integral in implementing AMU change, engendering considerations of interpersonal communication and hierarchy.^34^ This complex inter-professional practice environment means that the intent and motivation of one individual may not always be sufficient to successfully implement AMS change. AMS interventions relying on veterinarians as an initiator of change are therefore likely to benefit not only from developing veterinarian competence in principles of responsible prescribing, but also in developing their interpersonal skills (e.g. communication, empathy, conflict resolution, relationship building, motivating change) and providing AMS training to the wider inter-professional practice team, particularly where deficits in access to AMS CPD provision were also reported.

This AMS training approach could also enhance veterinarian interactions with their farm clients, who are collaborative stakeholders by acting as ‘recipients’ of veterinary AMS change (Table 4), whether explicitly within ‘farm oriented’ plans (e.g. as active participants in education and training provision or engaging in enhanced record keeping) or more implicitly in ‘practice oriented’ plans (e.g. as individuals navigating changed practice dispensing protocols). Challenges in motivating and sustaining change via the veterinary advisory role are well documented^35,36^ suggesting a focus on refined interpersonal skills may be crucial for veterinarians to engage in positive client interactions regarding AMS. Indeed, meta-ethnography suggests positive veterinarian-farmer interactions—embedded with a sense of trust and collaboration—are a key condition of the ‘enabling environment’ for veterinarians to feel able to pursue responsible prescribing practices.^37^

Implementation considerations regarding individuals and veterinary practice settings were also framed within complexities of the ‘outer setting’ environment, where participants discussed geographic, economic, regulatory, epidemiological and attitudinal challenges to AMS implementation. Most notably, VPC investment in AMS activity was articulated alongside concern for the lack of regulatory control on industry behaviours ‘*because the chain is only as strong as its weakest link’.* Client capacity to source AMs from multiple practices—whilst still falling within RCVS ‘under care principles’—ensured VPCs and other practice veterinarians could not be confident that they were the sole prescriber of AMs to a herd or flock. This eroded confidence in behaviour-led initiatives, where veterinarians expressed uncertainty on whether clients would simply obtain AMs from another veterinary practice rather than engage with AMS-led change. This uncertainty also weakened trust in the veterinarian-client relationship,^38^ highlighted previously as a key condition of the ‘enabling environment’^37^ for veterinarian AMS activity. The accuracy of structural initiatives was also impacted, where key sales records at one practice were felt to not necessarily be representative of the AMU on a specific farm. These structural initiatives were further complicated by broader complexities of benchmarking metrics^39^ (Table 6).

Data suggest the extent to which this regulatory concern was felt as a day-to-day AMS challenge by participants was determined by the partnerships and connections established between veterinary practices. This concern was most prominent for participants experiencing a lack of local between-practice communication—and thus minimal transparency of local prescribing relationships—in combination with experiences suggesting local practice prescribing cultures may conflict with their own (e.g. finding AMs in client medicine cabinets they would not prescribe themselves). In turn, the very geography of the practice setting may determine this experience, changing the nature and likelihood of neighbouring practice interactions. Research examining the spatial proximity, geography and comparative commercial activity of veterinary practices in Wales and the UK is currently lacking. However, Location Theory^40^—a group of theories that seek to explain the geographic positioning of economic activities- suggests this information could be valuable to target effective AMS support at a profession level by offering insight into how veterinary practices establish costs, what shapes community-practice interactions and how competitive dynamics emerge in local geographic contexts.^41^ This informed understanding could also enable more nuanced AMS support akin to human health services (e.g. NHS), where medicine use can be analysed, understood and targeted via well-characterized organizational structures and the expected interactions between prescribers and patients within them.^42^ Similarly, contextualized and targeted AMS messaging—responsive to local environmental, social, epidemiological and economic realities—could enhance AMS across Welsh veterinary practices.

Overall, this study highlights important considerations for interventions seeking to foster champion-led AMS change. By the nature of this (voluntary) national intervention, any influence on the variable prescribing landscapes experienced by the participants was only possible via scaffolding and supporting autonomous participant activity. Further interventions of this kind may benefit from considering several core questions arising from this study, which may help target intervention support effectively. Within the Arwain intervention, these questions have prompted responsive adjustments as it continues to underpin veterinary AMS across Wales (Table 7).

**Table 7.** Core intervention support questions prompting responsive adjustments within the Arwain complex intervention

Intervention foci	Questions to target intervention support	Arwain intervention adjustment
Goal setting	Are participants suitably skilled and informed in goal setting processes? Can evidence-based participant support improve articulation of change goals?	Renewed AMS goal-setting approach incorporating multiple levels of abstraction and educational support in understanding goal setting theory
Individuals	What support, education and training is available for wider AMS deliverers to facilitate champion-led AMS? What activity may be feasible to shape an allied ethos and culture within the ‘inner setting’?	Arwain provision of training and AMS support for full practice team through recruiting AMS ‘clinical leads’ Development of an ‘Arwain App’ for accessible AMS information Participatory development of clinical guidelines for six key diseases in cattle and sheep, relevant to both practice team and farm clientele
Inner setting		
Outer setting	What challenges are there in the wider prescribing landscape that may influence AMS? What appetite is there for participant-led solutions to counteract these challenges?	Participatory development of a voluntary national Arwain ‘Code of Conduct’ for responsible prescribing across Wales The development of voluntary between-practice Arwain AMU benchmarking activity

### Study considerations

A key limitation of this study is that data gathered on AMS achievements and challenges is self-reported by VPCs for their practices, which carries the risk of response bias due to perceived social and moral responsibility.^43^ As the research questions at the heart of this paper relate to veterinarian self-directed ‘AMU activity’—i.e. whether the Arwain intervention prompted VPC-led AMS change within veterinary practices—self-report as a data source is unavoidable, as knowledge on AMS changes made can only be reported by the VPCs themselves. Indeed, self-reported data is consequently a commonly used methodology in reviewing AMR/AMS interventions in the animal sector.^43^

Triangulating the achievement data reported in this study with quantitative measures of AMU would inform and strengthen the conclusions. Additionally, this study measured all AMS changes as single units to allow for analysis across all practices, which does not allow for the variable complexity and varying abstractness of the change goals recorded. Decision making within the code attribution process was however internally validated by the participating veterinarians themselves; each Arwain VPC reviewed the AMS change goal codes allocated to their Practice Action Plan in the process of the implementation review at the May Forum 2024.

### Conclusions

When given autonomy to design and implement practice-specific AMS change goals, participants focused on behaviour-led (practice team or farm client) and structural (farm- or vet practice-focused) changes. All Arwain practices reported at least one change goal initiated, with the majority of practices reporting one fully implemented change. AMS change goals varied in their abstractness and thus amenability to implementation review, suggesting a focus on evidence-based goal-setting practices would be beneficial in future veterinary AMS interventions. AMS implementation challenges included practical and cultural considerations of veterinary practices, the complexities of delivering AMS within inter-professional teams, the situated complexity of AMS on farm and the geographic, economic, regulatory, epidemiological and attitudinal factors implicit in the practice ‘outer setting’. Further research into the impact of these AMS changes on AMU is needed to evaluate and inform future policy on AMS.

## Supplementary Material

dlaf181_Supplementary_Data
