# Supplementary material for: Fostering behaviour change in farm veterinary practice: ‘champion’ goal-setting and implementation considerations for antimicrobial stewardship
Source: JAC Antimicrob Resist. 2025 Oct 24;7(5):dlaf181. doi: 10.1093/jacamr/dlaf181 (PMC12551451; doi:10.1093/jacamr/dlaf181)
Supplement: dlaf181_Supplementary_Data [file dlaf181_supplementary_data.docx]

# Supplementary Materials 1 – Qualitative Interview Schedules

Qualitative Interview Schedule: Arwain DGC

**Guidance**

Questions for each length of interview detailed below, designed by time limitation:

1. 15-minute: **essential**
2. 30-minute: **essential** and **desirable**
3. 45-60 minute: **essential** and **desirable (1^st^)**/**desirable(2^nd^)**

Questions are colour coded, to be adjusted based on the person interviewing.

*e.g. bring in some* ***desirable*** *questions to someone giving very short answers in a 15 min interview/scale back the questions to make sure you cover the* ***essential*** *questions in a very talkative 30 min interview*.

Progress interviews: 15 minutes

**Intro**

- Tell me a little about you and your practice
- What interested you about the AVC project/why did you put yourself forward?

**Intervention experience**

- How did you find the activities within the AVC project? (webinars, discussion groups, workshops) (prompt: feelings about online, in-person, blended- impact?)

**Actions**

- What actions did you intend to implement in your practice? (*interviewer: check sharepoint before completing the interview)*

**Stewardship experience**

- How have you found pursuing your intervention goals over the last 6 months?
- What have been your biggest successes? (and perceived impact of these?)
- What would you describe as the challenges in this process?
- What has surprised you?

**Identity**

- What does it mean to you to be a ‘Prescribing Champion’?

**Going forward**

- What would you like next from AVC?
- Are you involved in any other stewardship activities going forward?

*What else would you like to share that you feel is important?*

Progress interviews: 30 minutes

Questions: 15 mins

Extra questions: 30 mins

**Intro**

- Tell me a little about you and your practice
- What interested you about the AVC project/why did you put yourself forward?

**Interest in project/ intervention experience**

- How did you find the activities within the AVC project? (webinars, discussion groups, workshops)
- What worked/didn’t work in an online space?
- What changes would you make to the Arwain Vet Cymru project if we could re-run it?

**Actions**

- What actions did you intend to implement in your practice? (*interviewer: check sharepoint before completing the interview)*
- What motivated you to choose these actions?

**Stewardship experience**

- How have you found pursuing your intervention goals over the last 6 months?
- What have been your biggest successes? (perceived impact of these?)
- What would you describe as the challenges in this process?
- What has surprised you?
- What impact do you feel these are having?

**Identity**

- What does it mean to you to be a ‘Prescribing Champion’?
- To what extent has being a ‘Prescribing Champion’ affected your experience or interests as a vet?

**Going forward**

- What would you like next from AVC?
- How can we help make that happen?
- Are you involved in any other stewardship activities going forward?
- Is there anything you’re planning to do next as a prescribing Champion?

*What else would you like to share that you feel is important?*

Progress interview: 45-60 minutes

Questions: 15 mins

Extra questions: 30 mins

Extra questions: 45- 60 mins

**Intro**

- Tell me a little about you and your practice
- What led you into this career?
- What do you enjoy about your work?
- What are the challenges?
- What interested you about the AVC project/why did you put yourself forward?

**Interest in project/ intervention experience**

- How did you find the activities within the AVC project? (webinars, discussion groups, workshops)
- How did you feel about the activities being moved online, due to Covid-19?
- What worked/didn’t work in an online space?
- How would you prefer future meetings within AVC to be run? (Online, in person, blended) – why?
- What changes would you make to the Arwain Vet Cymru project if we could re-run it?
- What felt most valuable to you about your involvement in Arwain Vet Cymru?

**Actions**

- What actions did you intend to implement in your practice? (*interviewer: check SharePoint before completing the interview)*
- What motivated you to choose these actions?
- Were there any actions you wanted to take on, but felt unable to at this time? (Tell me more about that…)

**Stewardship experience**

- How have you found pursuing your intervention goals over the last 6 months?
- What have been your biggest successes? (perceived impact of these?)
- What would you describe as the challenges in this process?
- What has surprised you?
- What impact do you feel these are having?
- If you could rewind the clock, would you set out to tackle the same goals? (Tell me more about that…)

**Identity**

- What does it mean to you to be a ‘Prescribing Champion’?
- How would you describe the role and responsibilities of a Prescribing Champion within Welsh practices?
- To what extent has being a ‘Prescribing Champion’ affected your experience or interests as a vet?

**Going forward**

- What would you like next from AVC?
- How can we help make that happen?
- Are you involved in any other stewardship activities going forward?
- What do you think of these in contrast to AVC?
- Is there anything you’re planning to do next as a prescribing Champion? (Tell me more about that…)

*What else would you like to share that you feel is important?*

# Supplementary Materials 2

**Example content from Arwain Veterinary Prescribing Champions Action Plans 2021 and 2023, split by theme: (A) individual client behaviour; (B) oversight of farm antimicrobial use; (C) practice team behaviour; and (D) oversight of practice antimicrobial use.**

1. **Individual Client Behaviour**

Strategies aiming to influence client AMU on farm, whether by shaping and scaffolding (i) client autonomous behaviour on farm in the absence of the vet or (ii) decision making in partnership with the vet when both are on farm.

| **Category** | **Description** | **Example** |
| --- | --- | --- |
| **Education and training** | Educational activities carried out directly with the client(s) with the aim of sharing/exchanging information and upskilling | I would like to run some meetings to allow discussions and knowledge sharing regarding usage of antibiotics in calfhood disease and how to make responsible decisions on farm, as I feel that this is the most effective way of influencing changes.  Change goal: Host a farmer meeting to discuss calf disease management and treatment -advertisement in newsletter and targeted phone call invites to farmers that would be suitable. |
| **Access to information** | Increasing the availability and visibility of information freely available to clients relating to AMR/AMU | Engage with client community to communicate clearly and regularly AMR message and reasons behind any changes… Positive change with empowerment and engagement.  Change goal: Write newsletters. |
| **Refinement of AMU** | In partnership with the vet, adjusting client access to and use of AMs to encourage more responsible use | In house culture and sensitivity testing will save clients money and makes AB testing before prescribing more achievable for many and so allow a targeted and specific approach to AB use. Change goal: Promote in house culture and sensitivity testing amongst clients, set up introduction offers for clients to use culture and sensitivity testing. |
| **Alternatives to AMU** | In partnership with the vet, encouraging behaviours in place of AMU when appropriate | Want to increase proactive mindset and welfare and decrease antibiotic usage and reactive mindset.  Change goal: Increase in anti-inflammatory usage and decrease in long-acting antimicrobial usage. |
| **Prevention** | Working with the client to reduce their need for AMs on farm | By joining the Veterinary Prescribing Champions Network I hope to improve the standards of antimicrobial use within our practice, and the standards in which clients use and administer antimicrobials. The overall aim is to lower antimicrobial use and reduce the selection pressure for antimicrobial resistance.  Change goal: Focus disease management at prevention rather than treatment. |
| **Guidance/ instruction** | Scaffolding client behaviour to encourage responsible AMU | Standard protocols – practice and/or specific to farms – metaphylaxis in pneumonia, calf scours. Give us more control/more confidence in what prescription medicines are being used on farm and why.  Change goal: Each farm to have an on-farm protocol sheet for common conditions. Vet meeting to discuss/agree standardised protocols for e.g. calf pneumonia. Designate farms to particular vets. Start with dairies proactively. Move on to beef and sheep farms as we carry out their health reviews for farm assurance. Final phase would be to target non-farm assured beef and sheep farms. |

1. **Individual Client Behaviour**

Pursuing an effective working knowledge of farm management and/or AMU and using this knowledge/data in veterinary advice, communication and decision making

| **Category** | **Description** | **Example** |
| --- | --- | --- |
| **Monitoring** | Gathering detailed data on how and whether AMs and AMU guidance are being used on farms | Continue to monitor farmer purchases.  Change goal: Look at AMU calculations on FAWL and note high usage. Also monitor client purchases. Ask all team members to highlight any abnormal/unusual use. |
| **Auditing** | Structured review of AMU  or storage | Will add a medicine cupboard check to our health plans.  Change goal: Check medicine cupboards on health plans. Monitor correct storage of drugs, removal of out-of-date drugs, stress importance of correct handling and injection techniques. Support staff in charge of Health Plans to implement. Discussion with vets as to what to monitor/check for. |
| **Collating data** | Bringing together veterinary data to inform, structure or influence discussions with the client regarding AMU | Define limits for AB use on each farm.  Change goal: Sort farms out according to type of enterprise, then access AB audits and find average mg/PCU per enterprise. Use this to create targets for inclusion in annual HHP reviews. Tag onto HHPs, most clients are farm assured so already having an annual performance review. |
|  |  |  |
| **Targeting specific users** | Using veterinary knowledge and data to identify specific clients requiring further attention | Use the AMU calculator to quantify our client’s antimicrobial usage. We have recently gone through all farm clients and removed inactive accounts. I will use the AMU calculator to establish farms that potentially have issues with antibiotic usage so that we can work with these clients to rectify the issue…We are a newly established mixed practice (18 months since the practice was taken over) and currently have no grasp of antimicrobial usage by our farm clients. Before we can start addressing individual farms where there is a problem with antimicrobial usage, we need to establish which farms these are.  Change goal: Use the AMU calculator to quantify antibiotic usage on each farm… Make farmers aware of their own antibiotic usage and how this compares to other farms. |

**(C) Practice Team Behaviour**

Strategies aiming to shape and scaffold practice team advice giving, prescription and dispensing (specific to their role)

| **Category** | **Description** | **Example** |
| --- | --- | --- |
| **Training** | Educational activities carried out within the practice team with the aim of sharing/exchanging information and upskilling | Practice culture is the biggest problem for us: I feel this has to come first before education is then disseminated to farmers. It would be nice for change to be motivational rather than regulatory.  Change goals: Prescribing course to empower young vets: internal or external Organise training (external) for support staff  Signpost training (external) for younger vets: culture change/empower younger vets- Evidence-based Veterinary Medicine/medicines use |
| **Guidance/ instruction** | Scaffolding practice team behaviour to encourage responsible AMU | I am organising a meeting for all the vets to sit down and discuss the best treatment protocols for common FA diseases (calf pneumonia, calf scour, sheep and cow lameness etc). This way we can decide on most appropriate first line, second line etc treatment and all be on the same page when discussing cases like these with farmers.  Change goal: Producing treatment protocols for a selected number of diseases. |
| **Prescribing/ dispensing** | Shaping how and whether AMs are provided to clients by the practice upon request/prescription | Creating a flow chart for receptionist with a traffic light system for drugs they are allowed to book out for farmers without seeking veterinary advice (e.g. broad spectrum first line) and those more protected antibiotics that should only be dispensed after consultation with a veterinary surgeon regarding their intended usage.  Change goal: Restricting dispensing of protected antibiotics. |
| **Refinement of AMU** | Rules aiming to adjust practice team access to and use of AMs to encourage more responsible use | Planned change is to not permit the sale of large volumes of antibiotic or multiples doses of long-acting macrolides without work being carried out to investigate/improve management… It will hopefully reduce antibiotic sales.  It will educate farmers to improve management and re-educate them that antibiotics in place of good management is not acceptable. More vet time and work will replace revenue from lost sales.  Change goal: Not permitting sales of large volumes of antibiotic without investigation work or improved management. |
| **Promoting united voice** | Bringing the practice team together to foster collaboration and shared practice on AMU behaviours | Get vets together to make sure we are all singing from the same hymn sheet. Challenges include alternative beliefs and established norms, some understanding challenges down to distance from formal education.  Change goal: Specific meeting with vets and support staff regarding prescribing of antibiotics. |

1. **Oversight of Practice AMU**

Strategies aiming to shape and scaffold practice team advice giving, prescription and dispensing (specific to their role)

| **Category** | **Description** | **Example** |
| --- | --- | --- |
| **Monitoring** | Gathering detailed data on how and whether AMs are being used within the practice | Monitor the drugs purchased by individual clients more closely and relate it to the stock numbers. Establish what condition they are using the drugs for.  Change goal: Using computer software to monitor antimicrobial usage on each unit and encourage farmers to improve their record keeping. |
|  |  |  |
|  |  |  |
| **Auditing** | Structured review of AMU/dispensing/prescription | As we have recently expanded and taken on new staff, we need to introduce protocols to ensure consistency of our prescribing practices.  Change goal: Improve auditing of antibiotic use on beef farms and decide how we deal with farms that are not part of a Farm Assurance Scheme. |
|  |  |  |
| **Collating data** | Bringing together veterinary data to inform, structure or influence discussions with practice team regarding AMU | Look at practice sales of antibiotics… Animal sales data now useful as more stable client base- will give team better info on how we are doing.  Change goal: Look at practice sales of antibiotics- compare one year to previous and communicate to team… ask finance officer to ‘data bash’. |
|  |  |  |
|  |  |  |

# Supplementary Materials 3

Attribution of change goal codes to categories (i) across all coding Themes combined and (ii) within each separate coding Theme

** Indicates two most frequently allocated codes in 2020 and 2024*

| **Theme** | **Change goal category** | **All Themes combined: % attributed to code** | | **Within each theme: % attributed to code** | | **Within each theme: rank**  ***1 ‘most’ to 6 ‘least’ commonly attributed code*** | |
| --- | --- | --- | --- | --- | --- | --- | --- |
|  |  | **2020** | **2024** | **2020** | **2024** | **2020** | **2024** |
| Farm Client behaviour | Training | 11.5* | 17.5* | 32.7 | 48.6 | 1 | 1 |
|  | Access to information | 8.3 | 8.2 | 23.6 | 22.9 | 2 | 2 |
|  | Prevention | 7.7 | 5.2 | 21.8 | 14.3 | 3 | 3 |
|  | Refinement of AMU | 4.5 | 1.0 | 12.7 | 2.9 | 4 | 5 |
|  | Guidance/instruction | 1.9 | 3.1 | 5.5 | 8.6 | 5 | 4 |
|  | Alternatives to AMU | 1.3 | 1.0 | 3.6 | 2.9 | 6 | 5 |
| Farm oversight | Collating data | 7.7 | 6.2 | 19.4 | 40.0 | 1 | 1 |
|  | Targeting specific users | 3.8 | 5.2 | 16.1 | 33.3 | 2 | 2 |
|  | Monitoring | 4.5 | 4.1 | 12.9 | 26.7 | 3 | 3 |
|  | Auditing | 3.8 | 0.0 | 0.0 | 0.0 | 4 | 4 |
| Practice Team behaviour | Training | 9.6^x^ | 12.4^x^ | 21.4 | 33.3 | 1 | 1 |
|  | Promoting a united team voice | 5.1 | 9.3 | 16.1 | 25.0 | 2 | 2 |
|  | Dispensing | 7.7 | 5.2 | 8.9 | 13.9 | 3 | 3 |
|  | Refinement of AMU | 5.1 | 5.2 | 8.9 | 13.9 | 3 | 3 |
|  | Guidance/instruction | 8.3 | 5.2 | 8.9 | 13.9 | 3 | 3 |
| Practice oversight | Monitoring | 5.8 | 6.2 | 42.9 | 54.5 | 1 | 1 |
|  | Collating data | 0.6 | 4.1 | 28.6 | 36.4 | 2 | 2 |
|  | Auditing | 2.6 | 1.0 | 7.1 | 9.1 | 3 | 3 |
